# Analysis of Related Factors Influencing Hypertension Classification among Centenarians in Hainan, China

**DOI:** 10.31083/j.rcm2507235

**Published:** 2024-06-27

**Authors:** Jing Li, Jingfeng Bi, Shanshan Yang, Shengshu Wang, Shuwen Yang, Shimin Chen, Ke Han, Shengdong Luo, Qiyu Jiang, Miao Liu, Yao He

**Affiliations:** ^1^Department of Infectious Diseases Medicine, The Fifth Medical Center of Chinese PLA General Hospital, 100039 Beijing, China; ^2^Institute of Geriatrics, Beijing Key Laboratory of Research on Aging and Related Diseases, National Clinical Research Center for Geriatrics Diseases, 100853 Beijing, China

**Keywords:** centenarians, hypertension, healthy aging

## Abstract

**Background:**

As a population ages, blood pressure levels gradually increase, leading to a higher incidence of hypertension and increased cardiovascular diseases risk. This study examines factors affecting hypertension grading among centenarians in the Hainan Province.

**Methods:**

Data from 2014 to 2016 were accessed from the cross-sectional database “Hypertension Levels and Epidemiological Characteristics of the Elderly and Centenarians in Hainan province of China”. This study included 690 centenarians with hypertension. Hypertension grading was the dependent variable, analyzed against independent variables including demographic information (sex, age, ethnicity, education level, marital status, cohabitation, and regional distribution), lifestyle factors (smoking, alcohol consumption, and physical activity), body mass index (BMI), and comorbid conditions (diabetes and hyperlipidemia). Logistic regression models, adjusted for these factors, were used to assess the determinants of hypertension grading among the participants.

**Results:**

Multivariate regression analysis, after adjusting for other variables, revealed significant associations between BMI, low-density lipoprotein (LDL) levels, and hypertension grades. Individuals with BMI below 18.5 ${\mathrm{kg/m}}^{2}$ had a 0.614-fold lower risk of developing grade III hypertension (odds ratio [OR]: 0.614, 95% confidence interval [CI]: 0.390–0.966, *p* = 0.0350) and a 0.586-fold lower risk for grade II hypertension (OR: 0.586, 95% CI: 0.402–0.852, *p* = 0.0052). Furthermore, individuals with elevated LDL levels had a 6.087-fold greater risk of progressing from grade I to grade III hypertension (OR: 6.087, 95% CI: 1.635–22.660, *p* = 0.0071) and a 4.356-fold greater risk of progressing from grade II to grade III hypertension (OR: 4.356, 95% CI: 1.052–18.033, *p* = 0.0423). Additionally, individuals of Li ethnicity had 1.823-fold greater risk of progressing from grade I to grade II hypertension compared to those of Han ethnicity (OR: 1.823, 95% CI: 1.033–3.218, *p* = 0.0383).

**Conclusions:**

A BMI below 18.5 ${\mathrm{kg/m}}^{2}$, elevated LDL, and ethnicity emerged the primary factors associated with hypertension grading in centenarians. To reduce the risk of hypertension, it is crucial for centenarians to maintain a healthy weight, normal LDL levels, and adopt dietary habits including a low-cholesterol and low-fat diet.

## 1. Introduction

Promotion of healthy aging has become essential due to the rapid increase of aging in the Chinese population [1, 2]. Centenarians, representing the oldest segment of this demographic, serve as an ideal cohort for studying he dynamics of healthy aging due to their longevity [3, 4]. Notably, the incremental rise in blood pressure with advancing age has been observed in older adults [5], which correlates with an elevated prevalence of hypertension in this population. This trend exacerbates the risk of cardiovascular diseases [5, 6].

Previous studies established a significantly increase in the risk of cardiovascular disease among centenarians emphasizing the health challenges within this unique population [5, 6]. Notably, our analysis reveals pronounced disparities in hypertension risk based on both sex and geography. Specifically, female centenarians face a 1.624-fold higher risk of developing hypertension compared to their male counterparts, while individuals residing in the northern and central regions of the Hainan province are subject to a 0.625-fold higher risk than those in the eastern region [7]. To deepen our understanding of these disparities and other contributing risk factors, we utilized data from 2014 to 2016 to conduct a comprehensive epidemiological study on blood pressure levels within the centenarian population of Hainan, China. This study aims to elucidate critical factors influencing hypertension, thereby informing strategies for promoting healthy aging among the oldest adults.

## 2. Study Design and Methods

### 2.1 Population and Data Source

The data for this study were obtained from a cross-sectional research database that recorded the epidemiological characteristics and blood pressure levels of older adults and centenarians in the Hainan province of China. This comprehensive dataset encompasses the epidemiological survey data of a sample of 1002 centenarians (aged ≥100 years) residing in the Hainan province, collected between June 2014 and December 2016. Eligibility for inclusion required individuals to be alive during the specified period and traceable either through their addresses or family contacts. The dataset comprised a wide range of demographic information, including sex, age, ethnicity, educational background (illiterate, primary education, or above), marital status (married, widowed, divorced, or living alone), living arrangements (with family, living alone, or residing in elderly care institutions), and regional distribution (divided into eastern, western, southern, northern, and central regions of Hainan Province). Additionally, it records lifestyle habits (smoking, alcohol consumption, and physical exercise), body mass index (BMI), comorbidities (such as diabetes and cardiovascular diseases), and laboratory examination results (such as routine blood test and biochemical assays).

### 2.2 Data Analysis

#### Inclusion and Exclusion Criteria

Inclusion criteria were as follows: (1) Individuals 100 years or older, and (2) Hypertension, classified based on the Chinese Guidelines for the Prevention and Treatment of Hypertension (CGPTH-2018) which was revised in 2018 [8]. Exclusion criteria included the presence of tumors as well as cardiovascular and cerebrovascular diseases that may serve as triggering factors for hypertension.

### 2.3 Diagnostic Criteria for Hypertension

Blood pressure measurement: we dispatched a team of medical professionals to conduct blood pressure measurements at their homes. The resting blood pressure was measured using an upper arm electronic sphygmomanometer (Omron HEM-7200) with a precision level of 1 mmHg. Each subject’s blood pressure was recorded three times, with a three-minute interval between each measurement after they had sat down and rested for three minutes prior to the first reading.

Hypertension was characterized according to the CGPTH-2018, by systolic blood pressure (SBP) ≥140 mmHg and/or diastolic blood pressure (DBP) ≥90 mmHg, with grade I hypertension having SBP = 140–159 mmHg and/or DBP = 90–99 mmHg, grade II hypertension having SBP = 160–179 mmHg and/or DBP = 100–109 mmHg, and grade III hypertension having SBP ≥180 mmHg and/or DBP ≥110 mmHg. Based on the survey questions “Do you have hypertension?” and “Do you take medication for hypertension?”, when centenarians self-reported taking antihypertensive drugs or had a history of hypertension, and SBP ≥140 mmHg or DBP ≥90 mmHg, their hypertension classification was determined according to their blood pressure level. If they reported taking antihypertensive drugs or having a previous diagnosis of hypertension, but currently had SBP <140 mmHg and DBP <90 mmHg, they were classified as having grade I hypertension.

### 2.4 Definition of Severe Cardiovascular and Cerebrovascular Diseases

Severe cardiovascular diseases included myocardial infarction, aortic dissection, angina, heart failure, and arrhythmias. Severe cerebrovascular diseases included cerebral infarction, hypertensive intracerebral hemorrhage, subarachnoid hemorrhage, cerebral aneurysm, moyamoya disease, and cerebral vascular malformations.

### 2.5 Clinical Characteristics of the Study Population

The demographic information included gender, age, ethnicity, education level (illiterate, primary school, and above), marital status (married, widowed, divorced, or living alone), cohabitation status (living with family, living alone, or nursing homes), and regional distribution (Hainan province is divided into eastern, western, southern, northern, and central regions by administrative region). Habits included smoking, alcohol consumption, and exercise. Because almost all older males had a history of smoking and drinking at a certain time in the past, the specific time was not detailed owing to memory bias. None of the older females had a history of smoking or drinking, hence these variables were equivalent to sex.

To address this collinearity, smoking status was determined by the response to the questionnaire item “Do you smoke?”. Similarly, alcohol consumption status was determined by the response to the question “Have you consumed alcohol at least once in the past 12 months?”. This approach was also used to analyze the relationship between hypertension, smoking, and alcohol consumption. The extent of physical exercise was determined by the response to the question, “How many times per week do you engage in physical activities (such as housework and exercise)?”. BMI was calculated as weight/${\mathrm{height}}^{2}$ = ${\mathrm{kg/m}}^{2}$, and BMI <18.5, between 18.5 and 24, and ≥24 ${\mathrm{kg/m}}^{2}$ indicated underweight, normal weight, and overweight [9].

#### Concomitant Disease: Diabetes and Hyperlipidemia

The diabetes status was determined by inquiries regarding the patient’s medical history. Since most of the older individuals were not clear about whether they have hyperlipidemia, triglyceride (TG), cholesterol (TC), low-density lipoprotein (LDL) levels, and high-density lipoprotein (HDL) levels, values were obtained from laboratory tests during the investigation, and were used as analytical indicators to examine the relationship between hypertension and hyperlipidemia.

### 2.6 Statistical Analysis

Categorical data were classified according to their clinical significance. Age was categorized into two groups (≥105 and <105 years); BMI into three groups (<18.5, 18.5–24, and ≥24); and TC, TG, HDL, and LDL levels were dichotomized according to normal or elevated values. All categorical variables were expressed as percentages, and group comparisons were conducted using the chi-square test. Multiple logistic regression models employing dummy variables for multicategory independent variables were used to analyze the factors associated with hypertension of grades I, II, and III. All statistical analyses were performed using SAS 9.4 (SAS Inst. Inc., Cary, NC, USA), with a two-sided test and a significance level of *p* < 0.05.

## 3. Results

### 3.1 Study Population and Characteristics

**Fig. 1.**
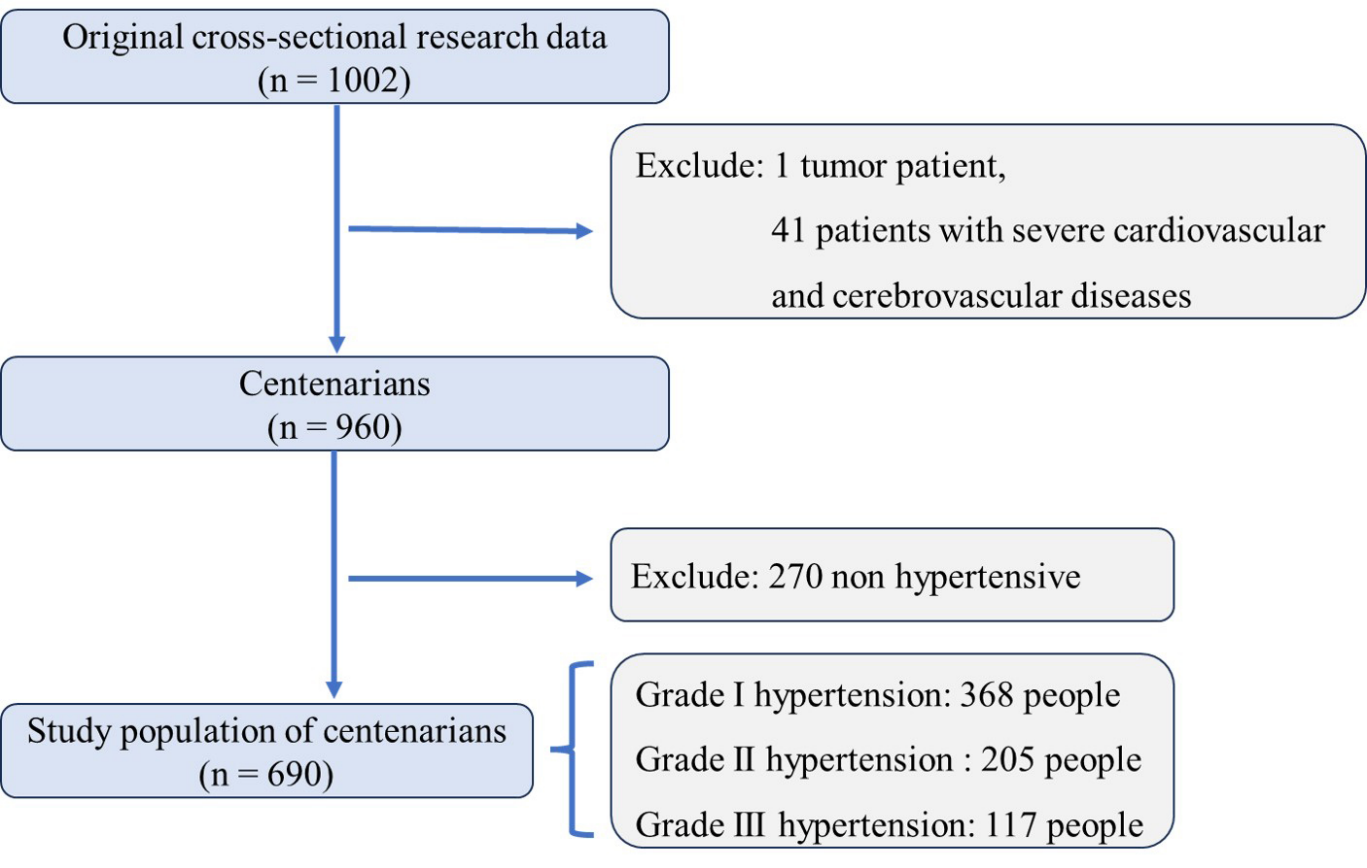
**Enrollment and selection criteria for centenarian participants in hainan hypertension study.** An overview of the enrollment and exclusion criteria applied to the initial sample of 1002 centenarians in our hypertension study. After excluding one participant with a tumor and 41 participants with severe cardio-cerebrovascular diseases, the final analysis included 960 participants. Among them, hypertension was prevalent, with 690 (71.88%) diagnosed with the condition. The figure also details the subdivision of hypertensive participants into different grades: Grade I hypertension was identified in 368 participants, Grade II in 205 participants, and Grade III in 117 participants, indicating the severity of hypertension across the study population.

In the original cross-sectional study, our sample comprised 1002 participants. To ensure the integrity of the data, exclusions were made for participants with confounding health issues: one individual diagnosed with a tumor and 41 individuals with severe cardio-cerebrovascular diseases, resulting in a final sample size of 960 centenarians. Analysis of this cohort revealed that hypertension was present in 690 participants, accounting for 71.88% of the sample, while the remaining 270 participants, or 28.12%, did not exhibit hypertension (Fig. 1).

**Fig. 2.**
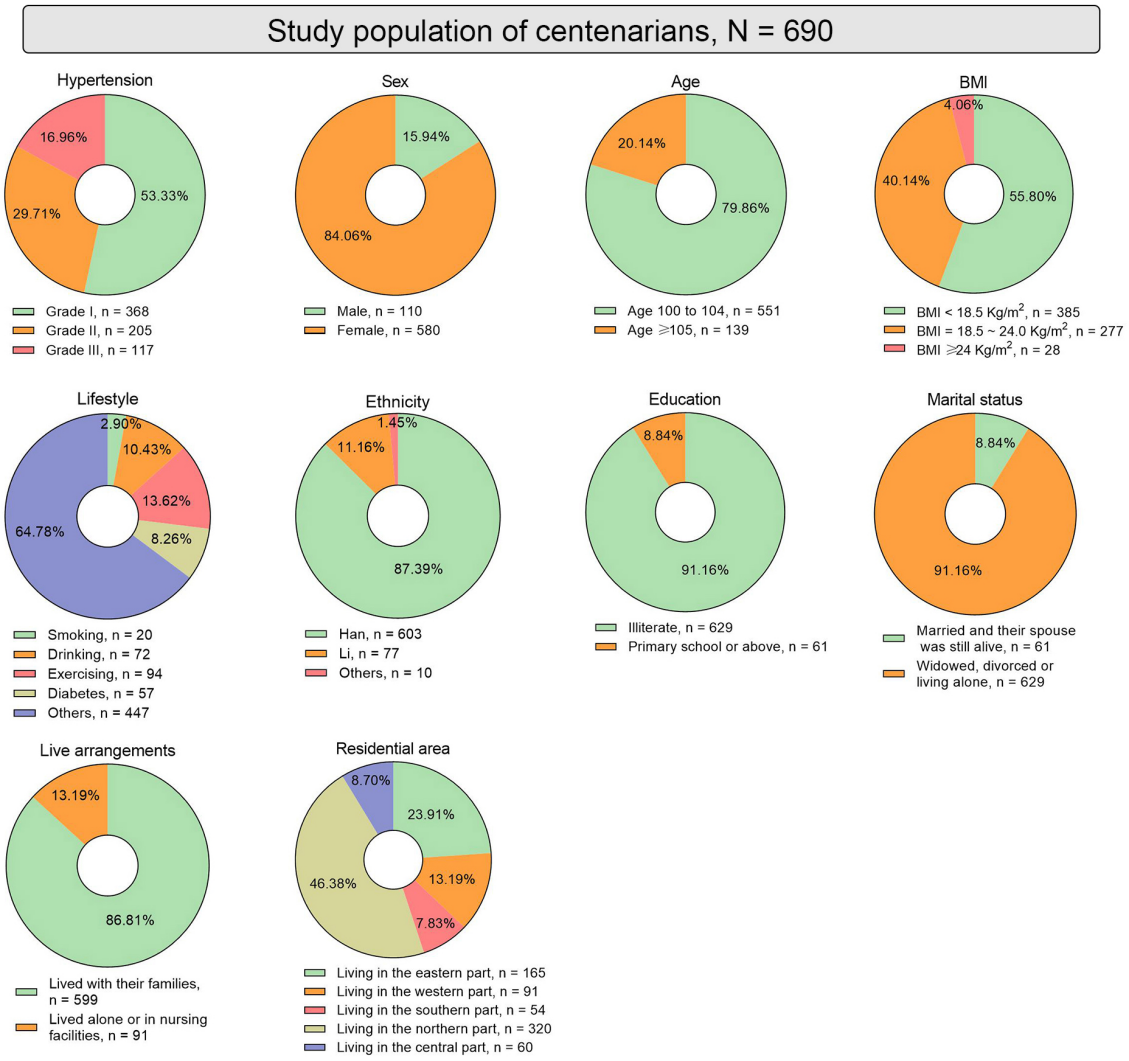
**Baseline characteristics of hypertensive centenarians in the Hainan province.** Fig. 2 illustrates the baseline characteristics of the hypertensive centenarian participants through multiple pie charts. These charts display the distribution of sex, age, BMI, lifestyle factors (smoking, alcohol consumption, and exercise frequency), diabetes prevalence, ethnic composition, educational levels, marital status, living arrangements, and geographic distribution within the Hainan province. BMI, body mass index.

Among the 690 hypertensive participants, the distribution of hypertension severity was as follows: 368 participants (53.33%) were classified with grade I, 205 (29.71%) with grade II, and 117 (16.96%) with grade III hypertension (Fig. 2). Demographic analysis revealed a significant sex disparity, 110 males (15.94%) and 580 females (84.06%) (Fig. 2). Notably, 551 participants were aged between 100–104 years (79.86%), while 139 were aged 105 years or older (20.14%) (Fig. 2). A total of 385 participants (55.80%) had a body mass index (BMI) of <18.5 ${\mathrm{kg/m}}^{2}$, 277 (40.14%) had a BMI within the normal range of 18.5 and 24 ${\mathrm{kg/m}}^{2}$, and only 28 participants (4.06%) had a BMI of 24 ${\mathrm{kg/m}}^{2}$ or above (Fig. 2). Lifestyle factors also varied within the cohort: 20 participants (2.90%) smoked, 72 (10.43%) consumed alcohol at least once in the past 12 months, and 94 (13.62%) engaged in regular physical activity at least once a week. Additionally, diabetes prevalence among the participants was 8.27%, with 57 individuals diagnosed. The majority of participants belonged to the Han (87.39%) and 77 Li (11.16%) ethnic groups. Educational levels were predominantly low, with 629 participants (91.16%) being illiterate, and only 61 (8.84%) having attended primary school or above. Marital status showed that 61 (8.84%) were married with surviving spouses, while the majority, 629 (91.16%), were widowed, divorced, or living alone. Living arrangements indicated that 599 participants (86.81%) resided with family, whereas 91 (13.19%) lived alone or in nursing facilities. Geographically, participants were spread across the Hainan province, with 320 (46.38%) in the northern region, 165 (23.91%) in the eastern region, 91 (13.19%) in the western region, 60 (8.70%) in the central region, and 54 (7.83%) in the southern region (Fig. 2).

### 3.2 Baseline Characteristics of 690 Older Participants with Grades I, II, and III Hypertension

A subgroup analysis was conducted with hypertension severity as the dependent variable to explore associations with various independent variables, which included demographic, lifestyle, and clinical factors such as age, sex, BMI, smoking status, alcohol consumption, physical activity, diabetes prevalence, and regional distribution. The results of this analysis are presented in Table 1. Despite the comprehensive range of factors analyzed, none of the independent variables showed a statistically significant association with the grades of hypertension among the participants.

**Table 1.** **Baseline characteristics of hypertensive centenarians in the Hainan province**.

Index		Grade I hypertension (n = 368)	Grade II hypertension (n = 205)	Grade III hypertension (n = 117)	*p* value
Sex, n (%)					
	Male	63 (17.12%)	33 (16.10%)	14 (11.97%)	0.4138
	Female	305 (82.88%)	172 (83.90%)	103 (88.03)	
Age, year, n (%)					
	100–105	297 (80.71%)	159 (77.56%)	95 (81.20%)	0.6165
	≥105	71 (19.29%)	46 (22.44%)	22 (18.80%)	
BMI, ${\mathrm{kg/m}}^{2}$, n (%)					
	<18.5	223 (60.60%)	100 (48.78%)	62 (52.99%)	0.0571
	18.5–24	131 (35.60%)	94 (45.85%)	52 (44.44%)	
	≥24	14 (3.80%)	11 (5.37%)	3 (2.56%)	
Smoking, n (%)		9 (2.45%)	7 (3.41%)	4 (3.42%)	0.7096
Drinking, n (%)		38 (10.33%)	23 (11.22%)	11 (9.40%)	0.8723
Physical exercise, n (%)					
	Sedentary	311 (84.51%)	171 (83.41%)	101 (86.32%)	0.7077
	≥1 time per week	52 (14.13%)	29 (14.15%)	13 (11.11%)	
	Unclear	5 (1.36%)	5 (2.44%)	3 (2.56%)	
Diabetes, n (%)		30 (8.15%)	19 (9.27%)	8 (6.84%)	0.7434
TC abnormal, n (%)		28 (7.61%)	14 (6.83%)	10 (8.55%)	0.8516
TG abnormal, n (%)		15 (4.08%)	10 (4.88%)	2 (1.71%)	0.3555
HDL abnormal, n (%)		41 (11.14%)	19 (9.27%)	7 (5.98%)	0.2517
LDL abnormal, n (%)		20 (5.43%)	12 (5.85%)	13 (11.11%)	0.0861
Ethnicity, n (%)					
	Han	327 (88.86%)	174 (84.88%)	102 (87.18%)	0.1091
	Li	39 (10.60%)	24 (11.71%)	14 (11.97%)	
	Other	2 (0.54%)	7 (3.41%)	1 (0.85%)	
Education, n (%)					
	Illiterate	335 (91.03%)	182 (88.78%)	112 (95.73%)	0.1067
	Primary school and above	33 (8.97%)	23 (11.22%)	5 (4.27%)	
Marital status, n (%)					
	Married	36 (9.78%)	17 (8.29%)	8 (6.84%)	0.5874
	Widow/Divorce/Living Alone	332 (90.22%)	188 (91.71%)	109 (93.16%)	
Live arrangements, n (%)					
	Living with family	321 (87.23%)	177 (86.34%)	101 (86.32%)	0.9420
	Living alone/Nursing facilities	47 (12.77%)	28 (13.66%)	16 (13.68%)	
Residential area, n (%)					
	East	90 (24.45%)	45 (21.95%)	30 (25.64%)	0.5720
	South	24 (6.52%)	21 (10.24%)	9 (7.69%)	
	West	55 (14.95%)	21 (10.24%)	15 (12.82%)	
	North	165 (44.84%)	99 (48.29%)	56 (47.86%)	
	Center	34 (9.24%)	19 (9.27%)	7 (5.98%)	

Abbreviations: BMI, body mass index; TC, total cholesterol; TG, triglycerides; HDL, high-density lipoprotein; LDL, low-density lipoprotein.

### 3.3 Multivariate Logistic Regression Analysis

We employed a multiple logistic regression model with multivalued nominal variables to analyze risk factors associated with different stages of hypertension—stages I, II, and III—among older participants. The outcomes, stratified by the severity of hypertension, are detailed across three tables (Tables 2,3,4). Table 2 compares hypertension Grade III with Grade I, Table 3 compares Grade III with Grade II, and Table 4 compares Grade II with Grade I.

**Table 2.** **Multivariate logistic regression analysis (hypertension grade III vs. I)**.

Variable	OR (95% CI)	*p*
Age, years (≥105 vs. 100–104)	0.955 (0.552–1.651)	0.8689
Sex (Male vs. Female)	1.465 (0.697–3.080)	0.3139
BMI, ${\mathrm{kg/m}}^{2}$ (<18.5 vs. 18.5–24)	0.614 (0.390–0.966)	**0.0350**
BMI, ${\mathrm{kg/m}}^{2}$ (≥24 vs. 18.5–24)	0.605 (0.161–2.266)	0.4554
Smoking (Yes vs. No)	2.215 (0.603–8.139)	0.2313
Drinking (Yes vs. No)	0.897 (0.409–1.966)	0.7863
Physical exercise (Sedentary vs. ≥1 time per week)	0.733 (0.373–1.442)	0.3684
Diabetes (Yes vs. No)	0.808 (0.351–1.857)	0.6152
TC elevated (Yes vs. No)	0.256 (0.065–1.007)	0.0511
TG elevated (Yes vs. No)	0.347 (0.071–1.701)	0.1920
HDL elevated (Yes vs. No)	0.484 (0.202–1.162)	0.1044
LDL elevated (Yes vs. No)	6.087 (1.635–22.660)	**0.0071**
Ethnicity (Li vs. Han)	1.600 (0.789–3.243)	0.1928
Education (Primary school and above vs. Illiterate)	0.537 (0.185–1.560)	0.2529
Marital status (Widow/Divorce/Living Alone vs. Married)	1.344 (0.577–3.127)	0.4931
Live arrangements (Living alone/nursing facilities vs. Living with family)	1.155 (0.606–2.201)	0.6618
Residential area (South vs. East)	0.876 (0.317–2.422)	0.7982
Residential area (West vs. East)	0.707 (0.327–1.532)	0.3799
Residential area (North vs. East)	0.977 (0.570–1.677)	0.9334
Residential area (Central vs. East)	0.402 (0.141–1.146)	0.0882
Residential area (West vs. South)	0.808 (0.294–2.221)	0.6791
Residential area (North vs. South)	1.116 (0.416–2.993)	0.8274
Residential area (Central vs. South)	0.459 (0.143–1.473)	0.1904
Residential area (North vs. West)	1.381 (0.671–2.844)	0.3804
Residential area (Central vs. West)	0.568 (0.198–1.628)	0.2923
Residential area (North vs. Middle)	2.433 (0.884–6.697)	0.0852

Bold *p*-values denote *p* < 0.05. Abbreviations: BMI, body mass index; TC, total cholesterol; TG, triglycerides; HDL, high-density lipoprotein; LDL, low-density lipoprotein; OR, odds ratio.

**Table 3.** **Multivariate logistic regression analysis (hypertension grade III vs. II)**.

Variable	OR (95% CI)	*p*
Age, years (≥105 vs. 100–104)	0.764 (0.425–1.374)	0.3693
Sex (Male vs. Female)	0.985 (0.438–2.216)	0.9713
BMI, ${\mathrm{kg/m}}^{2}$ (<18.5 vs. 18.5–24)	1.024 (0.626–1.674)	0.9257
BMI, ${\mathrm{kg/m}}^{2}$ (≥24 vs. 18.5–24)	0.487 (0.125–1.900)	0.3004
Smoking (Yes vs. No)	1.407 (0.352–5.620)	0.6293
Drinking (Yes vs. No)	0.788 (0.328–1.892)	0.5936
Physical exercise (Sedentary vs. ≥1 time per week)	0.740 (0.371–1.598)	0.4829
Diabetes (Yes vs. No)	0.691 (0.285–1.671)	0.4114
TC elevated (Yes vs. No)	0.381 (0.087–1.658)	0.1982
TG elevated (Yes vs. No)	0.354 (0.069–1.821)	0.2142
HDL elevated (Yes vs. No)	0.609 (0.237–1.568)	0.3044
LDL elevated (Yes vs. No)	4.356 (1.052–18.033)	**0.0423**
Ethnicity (Li vs. Han)	1.571 (0.590–4.180)	0.3659
Education (Primary school and above vs. Illiterate)	0.332 (0.110–1.003)	0.0506
Marital status (Widow/Divorce/Living Alone vs. Married)	1.094 (0.433–2.761)	0.8500
Live arrangements (Living alone/nursing facilities vs. Living with family)	1.057 (0.526–2.122)	0.8772
Residential area (South vs. East)	0.512 (0.175–1.495)	0.2207
Residential area (West vs. East)	0.949 (0.395–2.276)	0.9061
Residential area (North vs. East)	0.822 (0.453–1.493)	0.5203
Residential area (Central vs. East)	0.516 (0.166–1.605)	0.2529
Residential area (West vs. South)	1.854 (0.627–5.482)	0.2644
Residential area (North vs. South)	1.607 (0.572–4.516)	0.3680
Residential area (Central vs. South)	1.007 (0.296–3.430)	0.9905
Residential area (North vs. West)	0.867 (0.384–1.959)	0.7313
Residential area (Central vs. West)	0.543 (0.171–1.729)	0.3017
Residential area (North vs. Middle)	1.595 (0.534–4.766)	0.4029

Bold *p*-values denote *p* < 0.05. Abbreviations: BMI, body mass index; TC, total cholesterol; TG, triglycerides; HDL, high-density lipoprotein; LDL, low-density lipoprotein; OR, odds ratio.

**Table 4.** **Multivariate logistic regression analysis (hypertension grade II vs. I)**.

Variable	OR (95% CI)	*p*
Age, years (≥105 vs. 100–104)	1.249 (0.811–1.926)	0.3132
Sex (Male vs. Female)	1.489 (0.839–2.643)	0.1739
BMI, ${\mathrm{kg/m}}^{2}$ (<18.5 vs. 18.5–24)	0.586 (0.402–0.852)	**0.0052**
BMI, ${\mathrm{kg/m}}^{2}$ (≥24 vs. 18.5–24)	1.156 (0.486–2.752)	0.7425
Smoking (Yes vs. No)	1.816 (0.613–5.381)	0.2815
Drinking (Yes vs. No)	0.913 (0.492–1.694)	0.7737
Physical exercise (Sedentary vs. ≥1 time per week)	0.945 (0.564–1.584)	0.8311
Diabetes (Yes vs. No)	1.127 (0.605–2.099)	0.7069
TC elevated (Yes vs. No)	0.663 (0.228–1.923)	0.4491
TG elevated (Yes vs. No)	0.993 (0.411–2.399)	0.9884
HDL elevated (Yes vs. No)	0.778 (0.421–1.440)	0.4248
LDL elevated (Yes vs. No)	1.425 (0.444–4.577)	0.5517
Ethnicity (Li vs. Han)	1.823 (1.033–3.218)	**0.0383**
Education (Primary school and above vs. Illiterate)	1.591 (0.819–3.093)	0.1707
Marital status (Widow/Divorce/Living Alone vs. Married)	1.195 (0.632–2.261)	0.5834
Live arrangements (Living alone/nursing facilities vs. Living with family)	1.117 (0.657–1.900)	0.6818
Residential area (South vs. East)	1.711 (0.773–3.788)	0.1851
Residential area (West vs. East)	0.746 (0.385–1.446)	0.3849
Residential area (North vs. East)	1.188 (0.752–1.879)	0.4605
Residential area (Central vs. East)	0.779 (0.354–1.713)	0.5347
Residential area (West vs. South)	0.436 (0.192–1.012)	0.0573
Residential area (North vs. South)	0.694 (0.325–1.481)	0.3454
Residential area (Central vs. South)	0.455 (0.192–1.081)	0.0746
Residential area (North vs. West)	1.594 (0.867–2.931)	0.1338
Residential area (Central vs. West)	1.045 (0.460–2.372)	0.9163
Residential area (North vs. Middle)	1.525 (0.723–3.218)	0.2680

Bold *p*-values denote *p* < 0.05. Abbreviations: BMI, body mass index; TC, total cholesterol; TG, triglycerides; HDL, high-density lipoprotein; LDL, low-density lipoprotein; OR, odds ratio.

Our analysis revealed that the risk of developing grade III hypertension in participants with a low BMI (under 18.5 ${\mathrm{kg/m}}^{2}$) and grade I hypertension was significantly lower than that in those with a normal BMI (18.5 to 24 ${\mathrm{kg/m}}^{2}$) (odds ratio [OR]: 0.614, 95% confidence interval [CI]: 0.390–0.966, *p* = 0.0350) (Table 2). Similarly, the likelihood of progressing to grade II hypertension was also lower for participants with a low BMI (OR: 0.586, 95% CI: 0.402–0.852, *p* = 0.0052) (Table 4). However, there was no statistically significant difference in the risk of developing hypertension Grades I, II, or III among participants with a BMI above 24 ${\mathrm{kg/m}}^{2}$ compared to those within the normal BMI range (Table 2).

Additionally, our findings highlight a pronounced impact of LDL cholesterol levels on hypertension severity. Participants with elevated LDL levels and grade I hypertension have a significantly higher risk of developing grade III hypertension (OR: 6.087, 95% CI: 1.635–22.660, *p* = 0.0071) (Table 2). This was also observed in participants with grade II hypertension and elevated LDL (OR: 4.356, 95% CI: 1.052–18.033, *p* = 0.0423) (Table 3). No significant differences were observed between Grades II and I regarding LDL levels.

Ethnicity also played a role in hypertension risk, particularly between the Han and Li ethnic groups. Older individuals of Li ethnicity and grade I hypertension were at greater risk of developing grade II hypertension (OR: 1.823, 95% CI: 1.033–3.218, *p* = 0.0383) when compared to individuals of Han ethnicity (Table 4). However, no statistically significant differences in ethnicity were found between grades III and II hypertension or between grades III and I hypertension.

## 4. Discussion

The seventh National Population Census of China revealed a notable trend towards an aging society, with a significant correlation observed between aging and increased morbidity due to hypertension [10]. Among the older adult Chinese population, aged over 75 years, the rate of hypertension is expected to reach 60% [11]. Despite this, there is a scarcity of high-quality clinical studies targeting this population, inevitably adding to the difficulties in clinical decision making. In contrast, the Hainan province, often referred to as China’s ‘home of longevity’, reports a lower hypertension prevalence rate of 26.2% among adults—below the national average—possibly attributed to its unique natural environment and the dietary habits characterized by light flavors and low salt intake [12, 13]. Given these unique regional attributes, including centenarians from Hainan in this study offers a representative cross-section of the province’s diverse environments and genetic backgrounds, thereby providing valuable insights into the factors influencing hypertension in an aging population.

Numerous studies have established a robust link between obesity and hypertension, identifying BMI as a critical risk factor that influences both the likelihood and severity of hypertension [14, 15, 16]. Some studies suggest that maintaining an ideal BMI (20.0–23.9 ${\mathrm{kg/m}}^{2}$) and abdominal obesity (waist circumference ≥90 cm [male] and ≥85 cm [female]) are effective strategies for managing blood pressure [17, 18]. These findings are corroborated by our research, which demonstrates that individuals with a BMI below 18.5 ${\mathrm{kg/m}}^{2}$ were significantly less likely to progress from grade I to grade III hypertension, or from grade I to grade II hypertension compared to those with a normal BMI. However, our analysis did not reveal any significant differences in hypertension severity between individuals with a BMI >24 ${\mathrm{kg/m}}^{2}$ and those within the 18.5–24 ${\mathrm{kg/m}}^{2}$ range. This underscores the complex interplay between BMI and hypertension severity and highlights the need for further research to explore the thresholds at which BMI begins to significantly impact hypertension risk.

This study found that older adults with BMI below 18.5 ${\mathrm{kg/m}}^{2}$ had a lower risk of developing hypertension than those with a normal BMI, however no statistically significant differences were found in the hypertension rates between older adults with a normal BMI compared to older adults with an elevated BMI (**Supplementary Table 2**). The results indicate that the traditionally perceived “emaciated” status is a protective factor for centenarians with hypertension and severe hypertension grading. Our dataset indicates that individuals with a body mass index (BMI) less than 18.5 ${\mathrm{kg/m}}^{2}$ constitute 57.40% (551 individuals) of the sample (**Supplementary Table 1**), representing more than half of the centenarian population. Therefore, the traditional BMI classification may not be suitable for centenarians.

This study indicates that elevated LDL cholesterol substantially increases the risk of advancing to more severe hypertension stages among older adults. Specifically, individuals with elevated LDL levels and grade I hypertension were significantly more likely to progressing to grade III hypertension than that among those with normal LDL levels. This association between higher LDL levels and increased blood pressure supports existing literature [19]. Furthermore, LDL levels may be improved through physical exercise, maintaining a lower BMI, and increasing fiber intake [20].

Our study also revealed a role for ethnicity in hypertension. Particularly, the risk transitioning from grade I to grade II hypertension was significantly higher in the older population of Li ethnicity when compared to that of the Han ethnicity. This disparity may be influenced by lifestyle factors [21], particularly in the higher prevalence of smoking, alcohol consumption, and dietary preference for foods with high cholesterol and fat content [22]. Moreover, the lower educational attainment among older Li individuals often leads to inadequate attention to personal health and a lack of medical health knowledge, both of which contributes to the persistently elevated prevalence of chronic diseases such as hypertension [23].

There are researches and analysis of the factors influencing hypertension, found that female centenarians had a higher risk of hypertension [7]. This increased risk is thought to be associated with factors such as physiological hormone differences, cumulative lifestyle impacts, and sex-specific expression of longevity genes [1, 24, 25]. Notably, the decline in estrogen levels after menopause is believed to play a significant role [1, 24, 25]. Despite these findings, we did not identify a significant effect of sex on the severity grading of hypertension. This unexpected result suggests a complex interplay of factors at this advanced age and underscores the need for further research to elucidate the mechanisms behind these observations.

In this study analyzing factors influencing hypertension among 960 centenarians, we found the prevalence of hypertension among centenarians in the central and northern regions of the Hainan province was lower than that in other regions (**Supplementary Table 2**). This regional disparity may be attributed to environmental factors unique to these areas, such as higher altitude, dense forests, and large areas of tropical rainforests in the central and northern parts of Hainan which are thought to enrich atmospheric oxygen levels [26]. Such conditions could facilitate better regulation of blood pressure by the vasomotor center, potentially reducing the incidence of hypertension [26]. Despite these findings, we did not observe significant differences in hypertension classification by residential area. This suggests that other unexamined factors might influence the regional distribution of hypertension severity. Further investigation is needed to fully understand these dynamics and their implications for public health strategies.

Previous studies have consistently identified overweight and obese status, diabetes [27, 28], and lifestyle factors such as smoking, alcohol consumption, and lack of physical activity [8, 29] as primary risk factors for hypertension in adults. However, our findings did not establish a correlation between these traditional lifestyle factors—such as high BMI, diabetes, smoking, alcohol consumption, lack of exercise—and hypertension among centenarians. This discrepancy has been previously observed, and it has been suggested that the influence of traditional risk factors on hypertension may gradually decrease among centenarians [30, 31]. The phenomenon of aging itself could explain the attenuated relationship between these risk factors and hypertension, as it might modulate physiological responses [32]. Another possibility is that the longevity-survival effect evident in centenarian populations allows them to avoid or postpone the health impacts of risk factors and chronic diseases that lead to premature death, thus enabling this group to maintain good health and live longer lives [33]. Moreover, mental health factors, such as the association between diastolic dysfunction and depression identified by Tudoran *et al*. [34], may be relevant in certain cases. Therefore, the impact of psychological conditions on hypertension classification in centenarians deserves further investigation.

The elderly population is a unique group requiring distinct strategies for the prevention, diagnosis, evaluation, and treatment of hypertension, distinct from those used in the general population. Given their particular needs, it is crucial to tailor comprehensive hypertension management strategies for their needs, encompassing specific blood pressure targets, antihypertensive drug treatments, and lifestyle interventions. The STEP study underscores the effectiveness of hypertensive therapy, targeting a systolic blood pressure range of 110- to less than 130 mmHg, which significantly reduces the incidence of cardiovascular events in elderly hypertensive patients compared to standard therapy targeting a systolic blood pressure range of 130–150 mmHg [35]. This study provides important evidence-based support for future development of clinical management norms and guidelines for hypertension. However, there is currently insufficient evidence-based medical data specifically pertaining to centenarians. Given that most patients within this age group have poor overall health and multiple complications, individualized blood pressure control goals should be formulated based on their specific circumstances [36].

## 5. Conclusions

The prevalence of hypertension among centenarians in Hainan is notably high. Our findings indicate that the primary factors influencing hypertension classification in this population include a BMI below 18.5 ${\mathrm{kg/m}}^{2}$, elevated LDL levels, and ethnic differences. These insights suggest that maintaining a healthy weight to avoid emaciation, maintaining LDL cholesterol levels within normal ranges, and adopting dietary habits that emphasize a low-cholesterol and low-fat diet may have a positive impact on the management and prevention of hypertension.

This study is subject to several inherent limitations. First, all participants were recruited from the Hainan province, and although the dataset comprehensively represents this region, the results may not be generalized to centenarians nationwide or in other countries and territories. Second, the historical hypertension data and use of antihypertensive drugs were self-reported by the participants, introducing the potential for information bias. This could affect the accuracy of the data regarding hypertension prevalence and management.

## Data Availability

The data that support the findings of this study are available from the corresponding authors upon reasonable request.

## Supplementary Material

Supplementary material associated with this article can be found, in the online version, at https://doi.org/10.31083/j.rcm2507235.
