# Supplementary material for: Analysis of Related Factors Influencing Hypertension Classification among Centenarians in Hainan, China
Source: Rev Cardiovasc Med. 2024 Jun 27;25(7):235. doi: 10.31083/j.rcm2507235 (PMC11317323; doi:10.31083/j.rcm2507235)
Supplement: Supplementary file 1 [file 2153-8174-25-7-235-s1.docx]

**Supplementary materials**

**Analysis of influencing factors on hypertension in 960 centenarians**

The original database consisted of data from 1,002 centenarians. After excluding one patient with a tumor and 41 with serious cardiovascular and cerebrovascular diseases, the influencing factors of hypertension were analyzed in 960 qualified older adults.

Among the 960 centenarians, 690 (71.88%) had hypertension, while 270 (28.12%) did not. The study included 171 males (17.8%) and 789 females (82.2%). The age distribution showed that 760 individuals (79.2%) were aged between 100 and 104 years and 200 (20.8%) were aged ≥105 years. In terms of BMI, 551 participants (57.40%) had a BMI <18.5 kg/m², 373 (38.85%) had a BMI within the range of 18.5–24 kg/m^2^ and 24 (2.5%) had a BMI ≥24 kg/m². Additionally, 32 (3.3%) participants smoked, 99 (10.3%) drank alcohol at least once in the past year, 123 (12.8%) exercised once a week or more, and 90 (9.4%) had diabetes. The majority of the centenarians were of the Han and Li ethnicities, with 844 Han (87.9%) and 104 Li (10.8%) participants. Regarding education level, 877 participants (91.4%) were illiterate and 83 (8.6%) had an education level of primary school or above. 94 participants (9.8%) were married with their spouse still alive, while 866 (90.2%) were widowed, divorced, or living alone. In terms of living arrangements, 826 participants (86.0%) lived with their family members and 134 (14.0%) lived alone or in older adult care institutions. Geographically, according to administrative regions, 213 participants (22.2%) resided in the eastern part of Hainan province, 123 (12.8%) in the western part, 64 (6.6%) in the southern part, 468 (48.8%) in the northern part, and 92 (9.6%) in the central part.

**Comparison of baseline characteristics between 960 older individuals with hypertension and non-hypertension groups**

The older individuals were divided into two groups based on the presence of hypertension. The baseline data for the two groups are listed in Table S1. There were significant differences in sex (p = 0.0155) and residential area distribution (p = 0.0096) between the non-hypertension and hypertensive groups, whereas other variables were not statistically significant, indicating that hypertension in older adults may be related to sex and residential area.

**Supplementary Table 1. Comparison of baseline characteristics between 960 older patients with/without hypertension.**

| **Index** | | **Non hypertensive group**  **(n = 270)** | **Hypertension Group (n = 690)** | ***P* value** |
| --- | --- | --- | --- | --- |
| Sex, n (%) | |  |  |  |
|  | Male | 61 (22.59%) | 110 (15.94%) | **0.0155** |
|  | Female | 209 (77.41%) | 580 (84.06%) |  |
| Age, -year, n (%) | |  |  |  |
|  | 100-105 | 209 (77.41%) | 551 (79.86%) | 0.4011 |
|  | ≥105 | 61 (22.59%) | 139 (20.14%) |  |
| BMI, kg/m^2^, n (%) | |  |  |  |
|  | <18.5 | 166 (61.48%) | 385 (55.8%) | 0.2509 |
|  | 18.5-24 | 96 (35.56%) | 277 (40.14%) |  |
|  | ≥24 | 8 (2.96%) | 28 (4.06%) |  |
| Smoking, n (%) | |  |  |  |
|  | No | 258 (95.56%) | 670 (97.1%) | 0.2303 |
|  | Yes | 12 (4.44%) | 20 (2.9%) |  |
| Drinking, n (%) | |  |  |  |
|  | No | 243 (90%) | 618 (89.57%) | 0.8421 |
|  | Yes | 27 (10%) | 72 (10.43%) |  |
| Physical exercise, n (%) | |  |  |  |
|  | Sedentary | 239 (88.52%) | 583 (84.49%) | 0.222 |
|  | ≥1 time per week | 29 (10.74%) | 94 (13.62%) |  |
|  | Unclear | 2 (0.74%) | 13 (1.88%) |  |
| Diabetes, n (%) | |  |  |  |
|  | No | 237 (87.78%) | 633 (91.74%) | 0.0583 |
|  | Yes | 33 (12.22%) | 57 (8.26%) |  |
| TC abnormal, n (%) | |  |  |  |
|  | Normal | 256 (94.81%) | 638 (92.46%) | 0.1955 |
|  | High | 14 (5.19%) | 52 (7.54%) |  |
| TG abnormal, n (%) | |  |  |  |
|  | Normal | 264 (97.78%) | 663 (96.09%) | 0.1961 |
|  | High | 6 (2.22%) | 27 (3.91%) |  |
| HD abnormal, n (%) | |  |  |  |
|  | Normal | 234 (86.67%) | 623 (90.29%) | 0.1029 |
|  | High | 36 (13.33%) | 67 (9.71%) |  |
| LDL abnormal, n (%) | |  |  |  |
|  | Normal | 255 (94.44%) | 645 (93.48%) | 0.5782 |
|  | High | 15 (5.56%) | 45 (6.52%) |  |
| Ethnicity, n (%) | |  |  |  |
|  | Han | 241 (89.26%) | 603 (87.39%) | 0.6406 |
|  | Li | 27 (10%) | 77 (11.16%) |  |
|  | Other | 2 (0.74%) | 10 (1.45%) |  |
| Education, n (%) | |  |  |  |
|  | Illiterate | 248 (91.85%) | 629 (91.16%) | 0.7315 |
|  | Primary school and above | 22 (8.15%) | 61 (8.84%) |  |
| Marital status, n (%) | |  |  |  |
|  | Married | 33 (12.22%) | 61 (8.84%) | 0.113 |
|  | Widow/Divorce/Living Alone | 237 (87.78%) | 629 (91.16%) |  |
| Live arrangements, n (%) | |  |  |  |
|  | Living with family | 227 (84.07%) | 599 (86.81%) | 0.2712 |
|  | Living alone/Nursing facilities | 43 (15.93%) | 91 (13.19%) |  |
| Residential area, n (%) | |  |  |  |
|  | East | 48 (17.78%) | 165 (23.91%) | **0.0096** |
|  | South | 10 (3.7%) | 54 (7.83%) |  |
|  | West | 32 (11.85%) | 91 (13.19%) |  |
|  | North | 148 (54.81%) | 320 (46.38%) |  |
|  | Center | 32 (11.85%) | 60 (8.7%) |  |

Bold p-values denote p < 0.05. Abbreviation: BMI, body mass index; TC, total cholesterol; TG, triglycerides; HDL, high-density lipoprotein; LDL, low-density lipoprotein.

**Multivariate logistic regression analysis**

Using hypertension as the dependent variable, the independent variables in Table 1 were introduced into the logistic regression model. The results showed that after adjusting for other factors, the risk of hypertension in women was 1.669 times higher than that in men (p = 0.0194, OR: 1.669, 95% CI: 1.086 – 2.564). The risk of hypertension in older people with a BMI < 18.5 kg/m^2^ was 0.726 times lower than that in older people with a BMI of 18.5 – 24 kg/m^2^ (p = 0.0452, OR: 0.726, 95% CI: 0.531 – 0.993), while there was no significant statistical difference in hypertension between older people with a BMI of 18.5 – 24 kg/m^2^ and those with a BMI ≥ 24 kg/m^2^. The risk of hypertension in older adults with diabetes was 0.601 times lower than that in older adults without diabetes (p = 0.0344, OR: 0.601, 95% CI: 0.375 – 0.963). The risk of hypertension among older people living in the eastern region was 1.664 times higher than that among older people living in the northern region (p= 0.0111, OR: 1.664, 95% CI: 1.123 – 2.464) and 2.702 times higher than that among older people living in the central region (p = 0.0184, OR: 2.702, 95% CI: 1.131 – 3.798). The risk of hypertension among older people in the southern region was 2.499 times higher than that among older people in the northern region (p = 0.0190, OR: 2.499, 95% CI: 1.162 – 5.371) and 3.111 times higher than that among the older people in the central region (p = 0.0066, OR: 3.111, 95% CI: 1.372 – 7.054), as shown in Table S2.

**Supplementary Table 2. Multivariate logistic regression analysis.**

| **Variable** | **B** | **SE** | **OR (95% CI)** | **Wald/X2** | **P** |
| --- | --- | --- | --- | --- | --- |
| ≥105 vs. 100–104 | -0.0759 | 0.1801 | 0.927 (0.651–1.319) | 0.1776 | 0.6735 |
| Male vs Female | 0.5120 | 0.2191 | 1.669 (1.086–2.564) | 5.4627 | **0.0194** |
| BMI <18.5 vs. 18.5–24 | -0.3196 | 0.1596 | 0.726 (0.531–0.993) | 4.0094 | **0.0452** |
| BMI ≥24 vs. 18.5–24 | 0.3177 | 0.4327 | 1.374 (0.588–3.209) | 0.5391 | 0.4628 |
| Smoking Yes vs. No | -0.1616 | 0.4056 | 0.851 (0.384–1.884) | 0.1588 | 0.6903 |
| Drinking Yes vs. No | 0.0953 | 0.2688 | 1.100 (0.649–1.863) | 0.1257 | 0.7229 |
| Not exercising vs. ≥1 time per week | 0.3227 | 0.2347 | 1.381 (0.872–2.187) | 1.8899 | 0.1692 |
| Diabetes Yes vs. No | -0.5086 | 0.2405 | 0.601 (0.375–0.963) | 4.4727 | **0.0344** |
| TC abnormal Yes vs. No | 0.4363 | 0.4896 | 1.547 (0.593–4.039) | 0.7942 | 0.3728 |
| TG abnormal Yes vs. No | 0.6616 | 0.4804 | 1.938 (0.756–4.969) | 1.8963 | 0.1685 |
| HDL abnormal Yes vs. No | -0.4008 | 0.2367 | 0.670 (0.421–1.065) | 2.8673 | 0.0904 |
| LDL abnormal Yes vs. No | -0.4004 | 0.4821 | 0.670 (0.260–1.724) | 0.6899 | 0.4062 |
| Nation group: Li vs. Han | 0.1437 | 0.2547 | 1.155 (0.701–1.902) | 0.3184 | 0.5726 |
| Primary school and above vs. illiteracy | 0.3203 | 0.2963 | 1.378 (0.771–2.462) | 1.1689 | 0.2796 |
| Widow/Divorce/Living Alone vs. Married | 0.3324 | 0.2449 | 1.394 (0.863–2.253) | 1.8422 | 0.1747 |
| Living alone/in a nursing home vs. living with family | -0.0814 | 0.2124 | 0.922 (0.608–1.398) | 0.1468 | 0.7016 |
| South vs. East | 0.4065 | 0.4083 | 1.502 (0.674–3.343) | 0.9912 | 0.3194 |
| East vs. West | -0.1723 | 0.2798 | 1.188 (0.687–2.056) | 0.3793 | 0.5380 |
| East vs. North | -0.5092 | 0.2004 | 1.664 (1.123–2.464) | 6.4545 | **0.0111** |
| East vs. Central | -0.7285 | 0.3091 | 2.072 (1.131–3.798) | 5.5546 | **0.0184** |
| West vs. South | 0.5789 | 0.4138 | 1.784 (0.793–4.015) | 1.9568 | 0.1619 |
| West vs. North | -0.3368 | 0.2486 | 1.400 (0.860–2.280) | 1.8362 | 0.1754 |
| West vs. Central | -0.5562 | 0.3174 | 1.744 (0.936–3.248) | 3.0714 | 0.0797 |
| South vs. North | 0.9157 | 0.3905 | 2.499 (1.162–5.371) | 5.4986 | **0.0190** |
| North vs. Central | -0.2194 | 0.2791 | 1.245 (0.721–2.152) | 0.6176 | 0.4320 |
| South vs. Central | 1.1351 | 0.4176 | 3.111 (1.372–7.054) | 7.3867 | **0.0066** |

Bold p-values denote p < 0.05. Abbreviation: BMI, body mass index; TC, total cholesterol; TG, triglycerides; HDL, high-density lipoprotein; LDL, low-density lipoprotein.
